# Effects of Fish Oil on Biomarkers of Axonal Injury and Inflammation in American Football Players: A Placebo-Controlled Randomized Controlled Trial

**DOI:** 10.3390/nu14102139

**Published:** 2022-05-20

**Authors:** Veronica A. Mullins, Sarah Graham, Danielle Cummings, Alva Wood, Vanessa Ovando, Ann C. Skulas-Ray, Dennis Polian, Yiwei Wang, Gerson D. Hernandez, Claudia M. Lopez, Adam C. Raikes, Roberta D. Brinton, Floyd H. Chilton

**Affiliations:** 1 School of Nutritional Sciences and Wellness, University of Arizona, 1230 N Cherry Avenue, Tucson, AZ 85719, USA; vamullins@email.arizona.edu (V.A.M.); sarahgc2@email.arizona.edu (S.G.); dncummings@email.arizona.edu (D.C.); alvawood@email.arizona.edu (A.W.); ovandov@email.arizona.edu (V.O.); skulasray@arizona.edu (A.C.S.-R.); 2 Baylor Athletics, Baylor University, 1500 South University Parks Drive, Waco, TX 76706, USA; dennis_polian@baylor.edu; 3 Center for Innovation in Brain Science, University of Arizona, 1230 N. Cherry Avenue, Tucson, AZ 85719, USA; yvetteyww@gmail.com (Y.W.); gersonhe@arizona.edu (G.D.H.); claudiml@email.arizona.edu (C.M.L.); adamraikes@arizona.edu (A.C.R.); rbrinton@arizona.edu (R.D.B.)

**Keywords:** neurofilament light, DHA, football, subconcussive injury, head impacts, IL-6, TNF-a

## Abstract

There are limited studies on neuroprotection from repeated subconcussive head impacts (RSHI) following docosahexaenoic acid (DHA) + eicosapentaenoic acid (EPA) supplementation in contact sports athletes. We performed a randomized, placebo-controlled, double-blinded, parallel-group design trial to determine the impact of 26 weeks of DHA+EPA supplementation (n = 12) vs. placebo (high-oleic safflower oil) (n = 17) on serum concentrations of neurofilament light (NfL), a biomarker of axonal injury, and inflammatory cytokines (interleukin-6 (IL-6) and tumor necrosis factor-alpha (TNF-a)) in National Collegiate Athletic Association Division I American football athletes. DHA+EPA supplementation increased (*p* < 0.01) plasma DHA and EPA concentrations throughout the treatment period. NfL concentrations increased from baseline to week 26 in both groups (treatment (<0.001); placebo (*p* < 0.05)), with starting players (vs. non-starters) showing significant higher circulating concentrations at week 26 (*p* < 0.01). Fish oil (DHA+EPA) supplementation did not mitigate the adverse effects of RSHI, as measured by NfL levels; however, participants with the highest plasma DHA+EPA concentrations tended to have lower NfL levels. DHA+EPA supplementation had no effects on inflammatory cytokine levels at any of the timepoints tested. These findings emphasize the need for effective strategies to protect American football participants from the effects of RSHI.

## 1. Introduction

High-contact sports, such as football, involve a high risk of concussive injury and a higher risk of repeated subconcussive head impacts (RSHIs), impacts that do not meet the threshold for a concussion diagnosis [1,2,3]. Although the long-term effects of concussive injury have been associated with chronic traumatic encephalopathy [4], a progressive neurodegenerative disorder, the long-term effects of cumulative RSHI on brain structure, function, and mental health are unclear and a growing concern [2,5,6]. In American football, the number and magnitude of RSHIs have been associated with the age of onset of participation, position, time on the field, and style of play [7,8,9,10]. Studies in soccer and American football participants have reported increased serum neurofilament light (NfL), a biomarker of axonal injury, as a biomarker of RSHIs [11,12,13,14,15]. Interleukin-6 (IL-6) and tumor necrosis factor-alpha (TNF-a) are biomarkers of acute systemic inflammation previously reported to increase following traumatic brain injury [16] and mild traumatic brain injury (mTBI) from concussive injury in high school and collegiate football players [17]; however, questions remain regarding the relationship of serum IL-6 and/or TNF-a concentrations to RSHIs. In a 2019 position statement, the American Medical Society for Sports Medicine commented that RSHIs may create a risk of long-term neurological sequelae, and future research should include developing technologies that can assess brain changes following repetitive asymptomatic head trauma in living subjects [5].

The brain is highly enriched in long-chain (>20 carbon) highly polyunsaturated fatty acids (HUFAs) and docosahexaenoic acid (DHA; 22:6, omega-3 (n-3)). DHA is a primary structural and functional component of neuronal cell membranes; it regulates neurological processes involved in brain development and repair, including neuronal survival, synaptogenesis, myelination, neuronal plasticity, and neuroinflammation [18,19,20,21]. DHA deficiency leads to high omega-6 (n-6): n-3 HUFA ratios, particularly the ratio of arachidonic acid (ARA; 20:4, n-6) to DHA, and poor neurite growth and myelination and negative behavioral and mood symptoms in several animal models [20,22]. Eicosapentaenoic acid (EPA, n-3), which is present in much lower levels in the brain, produces bioactive mediators that are thought to reduce inflammation in the brain after injury [2,23,24].

DHA+EPA supplementation intervention studies in animal models have demonstrated neuroprotection after RSHIs [25,26], and a few clinical studies have examined the neuroprotective effects of DHA+EPA supplementation in NCAA American football participants [9,27]. Oliver et al. (2016) randomized 81 NCAA Division 1 football players to one of four groups, including 2, 4, or 6 g/day DHA supplementation or placebo for 27 weeks [9]. DHA supplementation at 2 g/day attenuated increased NfL levels in starters, with small-to-moderate effect sizes [9].

With limited research on the effects of fish oil supplementation on axonal injury from RSHIs in contact sports athletes, the primary aim of this study was to determine if DHA+EPA supplementation is effective in mitigating the biomarkers of adverse effects (axonal injury and inflammation) from RSHIs in collegiate American football athletes over the course of the football season. This study design provided a replication of the earlier 2016 study [9], with the addition of EPA to evaluate the effects of DHA+EPA supplementation on NfL; we also added an additional evaluation of the effects of supplementation on acute inflammatory biomarkers. Similar to Oliver et al. (2016), we evaluated the effects of start status; however, we also included analysis by position risk and provided DHA+EPA supplementation at one dose (3.5 g/day) vs. three (2, 4, 6 g/day) doses. We hypothesized that DHA+EPA supplementation would: (1) increase plasma DHA and EPA concentrations compared to placebo, (2) mitigate the increase in NfL serum concentrations in starters and high-risk groups, and (3) reduce IL-6 and TNF-a serum concentrations. 

## 2. Materials and Methods

All procedures involving human subjects were approved by the institutional review board of the University of Arizona for the use of human subjects in research (protocol 1904553365). Procedures were conducted in full accordance with the Declaration of Helsinki guidelines. All deidentified data are available at ClinicalTrials.gov #NCT0479207.

### 2.1. Participants

**Figure 1:**
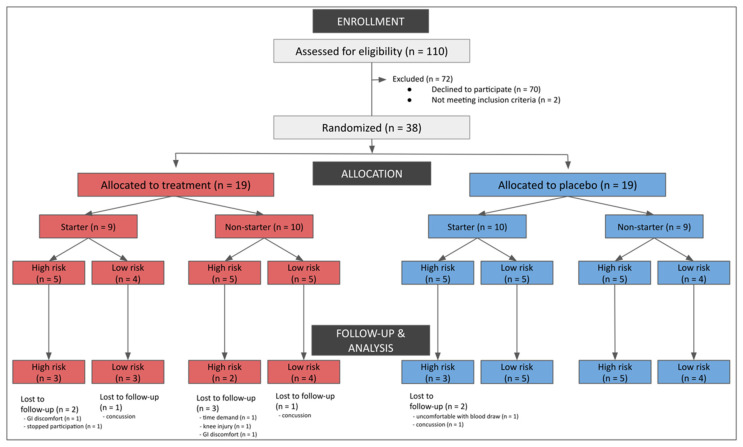
Consort diagram outlining reasons for exclusion.

National Collegiate Athletic Association Division I American football athletes cleared to participate in University of Arizona athletics (as determined by the team physician) were recruited by research personnel. Figure 1 includes a consort diagram outlining the reasons for exclusion. Participants were interviewed and excluded based on the following criteria: chronic daily anti-inflammatory use (≥20 days); antihypertensive medication use; lipid-lowering medication use; active fish oil or omega-3 fatty acid supplementation; consumption of more than two servings of fish per week; injured or unable to participate in regularly scheduled conditioning or competitions; and no diagnosed concussion at the time of enrollment. A total of 38 participants volunteered and provided written informed consent. Nine participants did not complete the study protocol (Figure 1). 

Adverse events are reported in Table 1.

### 2.2. Intervention Design

**Figure 2:**
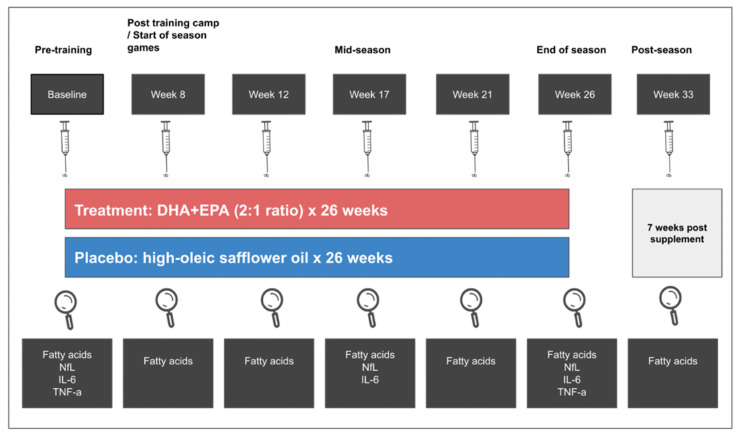
Schematic of the intervention design.

A schematic of the intervention design is illustrated in Figure 2. A randomized, double-blind, placebo-controlled, parallel-group design was employed. Treatment or placebo group assignment (1:1 for each group) was determined by a statistician using a block randomization method. Participants were stratified by start status (start and non-start) and risk by player position (high risk (offensive line, defensive line, running back, tight end, linebacker) and low risk (defensive back, special teams, wide receiver, quarterback)) [7]. Group assignment and randomization were recorded in a sealed envelope and blinded from researchers and study participants. Capsules were labeled (“A”, “B”) to maintain blinding, and both oils provided by Pharmavite (West Hills, California) were identical in appearance. Participants in the treatment group were required to take 3.5 g/d DHA+EPA (2.4:1.0 ratio) as ethyl esters in encapsulated form. Six 1 g soft gel capsules containing either 407 mg/g oil of DHA and 170 mg/g oil of EPA or 713 mg/g oil of oleic acid and 130 mg/g oil of linoleic acid from high-oleic safflower oil per capsule were provided 5 days per week for 26 weeks. Capsule fatty acid profiles are illustrated in Table S1. Supplement compliance was set at >80 percent for each subject and was assessed by research staff daily via visual supervision and counting returned/unconsumed capsules. Participants received their supplements from the research staff at the university training facility 5 days a week. If a participant did not consume the capsules, they were considered returned/unconsumed and returned to the lab, where they were counted and recorded. Whole blood was collected at baseline (before the beginning of non-contact conditioning) and then at weeks 8 (post summer training camp), 12 (regular season start), 17 (mid-season), 21, 26 (3–4 days after the last game), and 33 (7 weeks post end of season/treatment) (Figure 2).

### 2.3. Outcomes

The primary outcomes of this study were plasma fatty acid and serum NfL concentrations. Fatty acids were compared at all timepoints (baseline and weeks 8, 12, 17, 21, 26, and 33), while NfL was compared at three timepoints (baseline, week17, week 26). There were two secondary outcomes: first, a comparison of NfL concentrations between starters and non-starters and between low and high risks of exposure to RSHI positions; second, a comparison of interleukin 6 (IL-6) (baseline, week 17, week 26) and TNF-a (baseline, week 26) serum concentrations.

### 2.4. Biospecimen Collection

Biospecimens were collected by trained study personnel in a certified blood collection room at the football training facility, following all safety protocols. At each timepoint, 12 h fasted blood samples were collected from study participants by venipuncture into vacutainer tubes (4.5 mL tri-sodium citrate, 7.5 mL powdered glass clot activator, 10 mL ethylene diamine tetra-acetic acid (EDTA)) for a total of 22 mL of whole blood. Whole blood from the tri-sodium citrate tube was stored in coolers containing ice until sent to J2 laboratories (Tucson, AZ, USA) for analysis. EDTA vacutainer tubes were centrifuged at 3000 rpm for 15 min within 2 min of collection. The clot activator vacutainer tubes were allowed to clot for 30 min prior to being centrifuged at 3000 rpm for 15 min. Aliquots of serum (clot activator tube), plasma (EDTA tube), and red blood cells (EDTA tube) within the tubes were immediately transferred to polypropylene vials and stored at −80 ℃ until analysis. 

### 2.5. Plasma Fatty Acid Characterization

Fatty acid methyl esters (FAMEs) were prepared as previously described [28]. Plasma samples (100 μL) were added to tubes containing 25 μg of triheptadecanoin (C17:0) as internal standard and saponified with EtOH and 50% KOH at 60 °C for 30 min. The samples were acidified, and the fatty acids were extracted into hexane. Samples were brought to dryness under nitrogen, incubated for 5 min at 100 °C with 0.5 n NaOH in methanol, and FAMEs were prepared by incubation with 12% boron trifluoride for an additional 5 min at 100 °C. The FAMEs were extracted into hexane, brought to dryness under nitrogen, and resuspended in a small volume of isooctane for the GC analysis. The FAMEs were separated using a CP Select CB for FAME capillary GC column (100 m × 0.25 mm ID, film thickness 0.25 μm) and an HP 5890 temperature-programmed GC, with H2 as the carrier gas and helium as the make-up gas. The fatty acid distribution was determined via reference to the internal stand and analyzed by gas chromatography–flame ionization (GC–FID) using an Agilent Technologies 9000 gas chromatograph with an Agilent J&W DB-23 column (30 m, 0·25 mm ID, 0·25 µm film; Agilent) fitted with an inert pre-column (1 m, 0·53 mm ID) for cool on-column injection, following a protocol previously described [29,30].

### 2.6. Neurofilament Light 

Serum NfL concentrations (pg/mL) were determined by Quanterix Corp using the NF-Light Simoa Assay Advantage Kit (Quanterix Corp, MA, USA). 

### 2.7. Interleukin-6 and Tumor Necrosis Factor-Alpha

Serum IL-6 and TNF-a concentrations (pg/mL) were determined using ELISA (R&D Systems high-sensitivity kit (Minneapolis, MN, USA)).

### 2.8. Statistical Analysis

All analyses were performed using RStudio statistical computing software (RStudio, Version 1.3.1093 © 2009–2020 RStudio, PBC, Boston, MA, USA) together with R version 4.0.5 (R: A Language and Environment for Statistical Computing, R Core Team, R Foundation for Statistical Computing, Vienna, Austria, 2020). Natural logarithmic transformation was used for positively skewed outcome variables. The outcomes of interest were HUFAs, NfL, IL-6, and TNF-a. Linear mixed-effects models were used to model the effects of supplementation on those markers of interest by time, group, and time-by-group interactions. Models were fit using the lme4 [31] package, and hypothesis tests and estimated marginal means calculations were performed using the lmetest [32] and emmeans [33] packages. In the estimated marginal means analyses, *p*-values were adjusted using Tukey’s method. Effect sizes between treatment groups for NfL concentrations were computed using Cohen’s d and the devtools [34] and effectsize [35] packages. Effect sizes were interpreted as small (d = 0.2), medium (d = 0.5), and large (d = 0.8) based on standard benchmarks [36]. Correlations were calculated using Pearson’s Product Moment correlation formula using the ggpub package [37]; *p*-values < 0.05 were considered statistically significant for all analyses.

## 3. Results

### 3.1. Effects of Supplementation on Plasma Fatty Acid Concentrations

**Figure 3:**
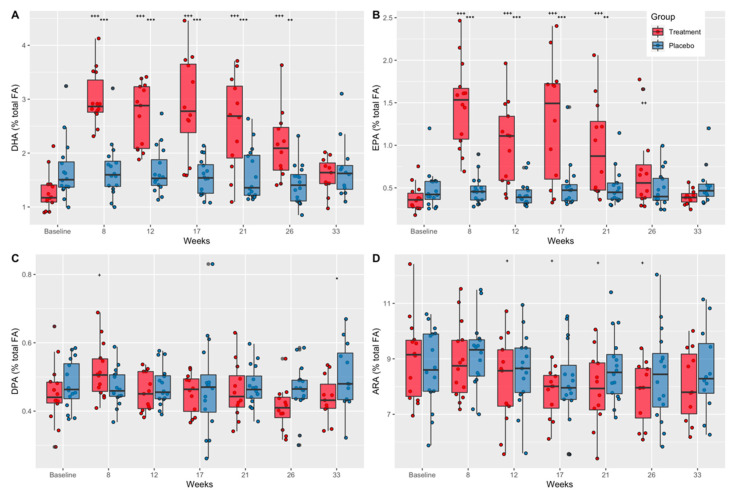
The effect of DHA+EPA supplementation on plasma fatty acid concentrations as a percent of total fatty acids. (**A**) DHA concentrations as a percent of total fatty acids in treatment and placebo groups from baseline through week 33. (**B**) EPA concentrations as a percent of total fatty acids in treatment and placebo groups from baseline through week 33. (**C**) DPA concentrations as a percent of total fatty acids in treatment and placebo groups from baseline through week 33. (**D**) ARA concentrations as a percent of total fatty acids in treatment and placebo groups from baseline through week 33. DHA, docosahexaenoic acid; EPA, eicosapentaenoic acid; DPA, docosapentaenoic acid; ARA, arachidonic acid; *p*-values show significance between groups (*) and within groups from baseline values (+) according to repeated measures ANOVA and estimated marginal means; *p*-values: * + *p* < 0.05; ** ++ *p* < 0.01, *** +++ *p* < 0.001.

Plasma fatty acid concentrations (expressed as percentages of total fatty acids) as a function of time and treatment group are shown in Table S2. ANOVA analysis indicated a significant difference in plasma concentrations across time and group for four HUFAs (DHA, EPA, docosapentaenoic acid (DPA), and ARA) (Figure 3).

#### 3.1.1. Docosahexaenoic Acid (DHA)

Further analysis indicated that plasma DHA concentrations were significantly greater than baseline in the treatment group at weeks 8, 12, 17, 21, and 26 (*p* < 0.0001), but returned to baseline values at week 33, 7 weeks after the end of treatment (Figure 3A). These results are consistent with compliance estimates from pill counts and indicate that compliance diminished slightly over time. There were no differences across time in plasma DHA concentrations in the placebo group. Plasma DHA concentrations in the treatment group compared to placebo were significantly less at baseline (mean diff. = −0.40, CI = 0.95, *p* = 0.048) and significantly greater at week 8 (mean diff. = 1.33, CI = 0.95, *p* < 0.0001), week 12 (mean diff. = 1.03, CI = 0.95, *p* < 0.0001), week 17 (mean diff. = 1.29, CI = 0.95, *p* < 0.0001), and week 21 (mean diff. = 0.92, CI = 0.95, *p* < 0.0001) and at week 26 (mean diff. = 0.68, CI = 0.95, *p* = 0.001) (Figure 3A).

#### 3.1.2. Eicosapentaenoic Acid (EPA)

EPA concentrations in the treatment group were significantly greater than baseline at week 8, 12, 17, and 21 (*p* < 0.0001), and week 26 (*p* < 0.01) (Figure 3B). There were no differences across time in plasma EPA concentrations in the placebo group (Figure 3B). Additionally, plasma EPA concentrations were greater in the treatment group compared to placebo at week 8 (mean diff. = 0.49, CI = 0.95, *p* < 0.0001) and at week 12 (mean diff. = 0.34, CI = 0.95, *p* < 0.0001), week 17 (mean diff. = 0.35, CI = 0.95, *p* = 0.004), and week 21 (mean diff. = 0.24, CI = 0.95, *p* = 0.001) (Figure 3B).

#### 3.1.3. Docosapentaenoic Acid (DPA)

Plasma DPA concentrations were significantly greater than baseline in the treatment group at week 8 (*p* = 0.003) and were significantly lower in the treatment group compared to placebo at week 33 (mean diff. = −0.07, CI = 0.95, *p* = 0.03) (Figure 3C). There were no differences across time in plasma DPA concentrations in the placebo group (Figure 3C).

#### 3.1.4. Arachidonic Acid (ARA)

Plasma ARA concentrations were significantly lower in the treatment group at week 12 (*p* = 0.046), 17 (*p* = 0.011), 21 (*p* = 0.03), and 26 (*p* = 0.010) compared to baseline (Figure 3D). There were no between-group differences in plasma ARA concentrations (Figure 3D).

#### 3.1.5. ARA:DHA and ARA:EPA Ratios

The ratios of ARA to DHA and ARA to EPA levels are shown in Table 2 and further highlight the shift in the balance of circulating n-6 versus n-3 LC-HUFAs in the treatment (DHA+EPA) group compared to placebo. The ARA:DHA ratio was reduced from 7:1 pretreatment to 3:1 in the treatment group at weeks 8, 12, 17, and 21 (Table 2).

### 3.2. Effects of Supplementation on a Marker of Axonal Injury (Treatment vs. Placebo)

**Figure 4:**
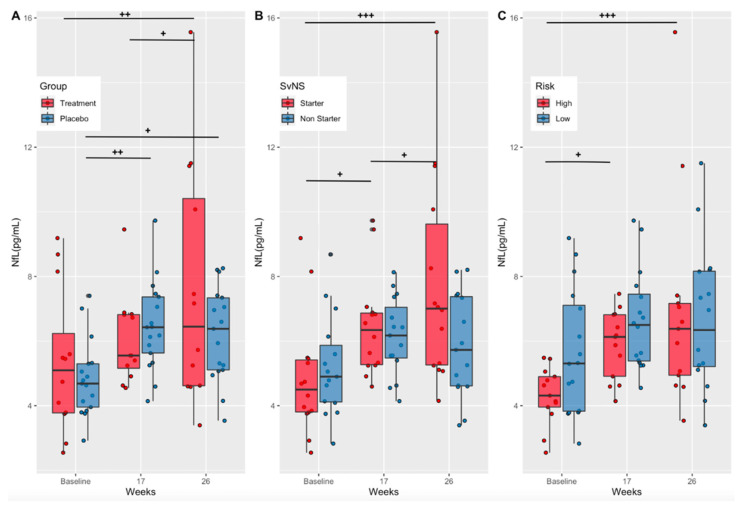
Serum NfL concentrations by treatment group, starter status, and risk. (**A**) Serum NfL concentrations (pg/mL) in the treatment and placebo groups at baseline, mid-season (week 17), and at end of treatment (week 26). (**B**) Serum NfL concentrations (pg/mL) in starters and non-starters at baseline, mid-season (week 17), and at end of treatment (week 26). (**C**) Serum NfL concentrations (pg/mL) in high- and low-risk position groups at baseline, mid-season (week 17), and at end of treatment (week 26). NfL, neurofilament light; *p*-values show significance within groups from baseline values (+) according to repeated measures ANOVA and estimated marginal means; *p*-values + *p* < 0.05; ++ *p* < 0.01, +++ *p* < 0.001.

Serum NfL concentrations (pg/mL) increased over time in both the treatment (Baseline-Week 26, *p* = 0.001; Week 17–26, *p* = 0.03) and placebo (Baseline-Week 17, *p* = 0.005; Baseline-Week 26, *p* = 0.02) groups, with no statistically significant differences between groups at any one timepoint (Figure 4A). Although not statistically significant, NfL levels in the treatment group were lower than the placebo group at week 17 (Cohen’s d = −0.31 (low–moderate effect size)) and higher at week 26 (Cohen’s d = 0.53 (moderate effect size)).

### 3.3. Effects of Playing Time and Position Risk on a Marker of Axonal Injury (Starters vs. Non-Starters and High-Risk vs. Low-Risk Positions)

As stated previously, the number and magnitude of RSHIs have been associated with time on the field and position [7,9]. Therefore, NfL concentrations were compared between starters and non-starters as well as between players in high- versus low-risk positions. Serum NfL concentrations (pg/mL) increased over time in starters (Baseline-Week 17, *p* = 0.0124; Baseline-Week 26, *p* < 0.001), with no significant increases seen in non-starters (Figure 4B). Starters exhibited significantly greater NfL levels at week 26 compared to non-starters (*p* = 0.0086) (Figure 4B). Further analysis indicated that participants in high-risk positions had a significant increase in NfL concentrations over time (Baseline-Week 17, *p* = 0.02; Baseline-Week 26, *p* < 0.001), with no change seen in low-risk positions. However, there were no significant differences between high- and low-risk groups at any time point (Figure 4C).

### 3.4. Effects on Inflammatory Cytokines

**Figure 5:**
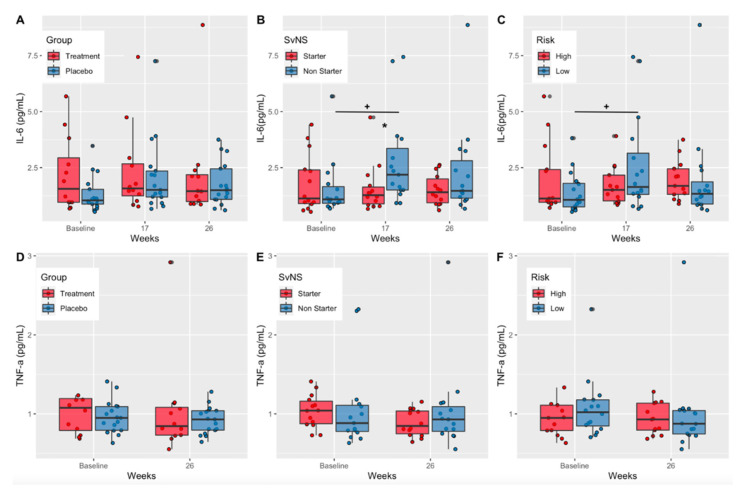
Serum IL-6 and TNF-a concentrations by treatment group, starter status, and risk. (**A**) Serum IL-6 concentrations (pg/mL) in the treatment and placebo groups at baseline, mid-season (week 17), and at end of treatment (week 26). (**B**) Serum IL-6 concentrations (pg/mL) in starters and non-starters at baseline, mid-season (week 17), and at end of treatment (week 26). (**C**) Serum IL-6 concentrations (pg/mL) in high- and low-risk position groups at baseline, mid-season (week 17), and at end of treatment (week 26). (**D**) Serum TNF-a concentrations (pg/mL) in the treatment and placebo groups at baseline, mid-season (week 17), and at end of treatment (week 26). (**E**) Serum TNF-a concentrations (pg/mL) in starters and non-starters at baseline, mid-season (week 17), and at end of treatment (week 26). (**F**) Serum TNF-a concentrations (pg/mL) in high- and low-risk position groups at baseline, mid-season (week 17), and at end of treatment (week 26). IL-6, interleukin-6; TNF-a, tumor necrosis factor-alpha; *p*-values show significance within groups from baseline values (+) according to repeated measures ANOVA and estimated marginal means; *p*-values + *p* < 0.05.

IL-6 and TNF-a are acute inflammation biomarkers elevated in moderate-to-severe cases of mTBI [17,38,39]; however, studies focused on IL-6 and TNF-a in RSHIs are lacking. There were no significant changes in serum IL-6 concentrations over time or between treatment groups (DHA+EPA vs. safflower oil placebo) (Figure 5A). When comparing serum IL-6 concentrations in starters v. non-starters, concentrations increased significantly in non-starters from baseline to week 17 (*p* = 0.02); they had significantly elevated concentrations compared to starters at week 17 (*p* = 0.02). (Figure 5B). Additionally, low-risk positions had significantly higher IL-6 concentrations compared to high-risk positions at week 17 (*p* = 0.04) (Figure 5C). There were no time or group differences in serum TNF-a concentrations (Figure 5D–F).

### 3.5. Associations between DHA+EPA and NfL Concentrations

**Figure 6:**
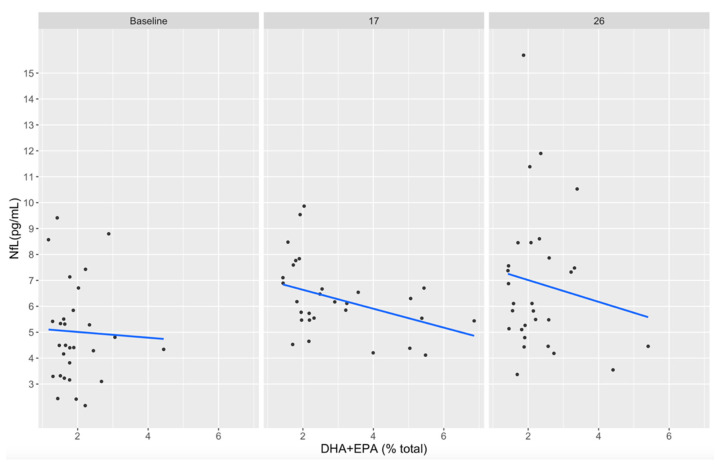
Correlation between plasma DHA+EPA (% total fatty acids) and serum NfL (pg/mL) concentrations in treatment and placebo groups at baseline, week 17, and week 26. DHA, docosahexaenoic acid; EPA, eicosapentaenoic acid; NfL, neurofilament light.

Individual participant plasma EPA+DHA concentrations did not reach a level of >8% total fatty acids (the current standard for adequate consumption) at any timepoint. However, plasma DHA+EPA concentrations appeared inversely associated with serum NfL concentrations at week 17 (r = −0.42, *p* = 0.17) and week 26 (r = −0.44, *p* = 0.15), although these observations were not statistically significant (Figure 6). This is not surprising given the small within-group sample size but is reported here for planning future studies.

## 4. Discussion

Animal and human studies have demonstrated that DHA+EPA supplementation reduces the n-6/n-3 HUFA ratio and provides neuroprotective effects (reduced axon swelling and breakdown, neuroinflammation, axonal injury, and oxidative stress) from RSHIs [11,19,25,26]. Collegiate and elite athletes often have low circulating plasma DHA and EPA concentrations directly related to poor dietary intake of these n-3 HUFAs [40,41], which may contribute to short- and long-term sequelae associated with RSHIs. The current study examined the effects of fish oil (3.4 g/day DHA+EPA) supplementation as ethyl esters on markers of axonal injury (NfL) and acute inflammation (IL-6, TNF-a) from RSHIs in NCAA Division 1 football players. Circulating plasma DHA and EPA concentrations increased 2–3-fold (Figure 3A,B) and n-6/n-3 HUFA ratios were reduced (Table 2), validating previous fish oil supplementation (DHA+EPA) studies [42,43].

NfL has been utilized to assess sports-related mTBIs because it is a neurological biomarker that can be measured in the blood and because levels remain elevated after injury [13]. Neurofilaments are components of the neuron cytoskeleton. Following mTBI, calcium influx into the cell contributes to a cascade of events that dephosphorylates neurofilament side-arms, contributing to axonal injury [44]. In the current study, serum NfL levels increased significantly, without a concussion diagnosis, in the treatment, placebo, starters, and high-risk position groups (Figure 4A–C) and were significantly higher in starters versus non-starters at the end of the season (Figure 4B). These results could be due to increased playing time (starters) and/or an increase in RSHIs in the starters and high-risk position players. Although not measured directly as part of this study, the number of head impacts per season has been shown to be greater in players with the greatest on-field participation during competition [1]. Fish oil supplementation did not mitigate the increase in NfL in the treatment group compared to placebo. Therefore, we were unable to replicate the previously observed decrease in serum NfL with DHA+EPA oral supplementation when compared to the placebo seen by Oliver et al. (2016), possibly due to lower NfL values overall in this study (2.3–15.5 pg/mL vs. 3.7–23.8 pg/mL), indicating less axonal injury [9]. In healthy adults, serum NfL levels range from 2.8 to 9.7 pg/mL [45]. NfL levels post heading in soccer have been measured at 16.1 pg/mL [14] and as high as 26 pg/mL 10 days post sports-related concussion [13].

IL-6 and TNF-a are biomarkers of acute inflammation peaking within 24 h post sports-related concussion [16,38]. Several studies have reported elevated IL-6 immediately after mTBI in American football participants [17,38,39]; however, measurements of IL-6 in serum may be more indicative of peripheral injury or BBB integrity in this circumstance [16]. In the present study, IL-6 levels exhibited little variation between timepoints, with a significant increase in non-starters and low-risk positions from baseline to week 17. This likely reflects activity 24 h prior to the blood draw and not gameday activity or RSHIs (Figure 5A–C). Due to football scheduling issues, blood collection within 24 h following game participation was not possible in the current study. Consequently, the timing of the blood draws (3–4 days post game day) was a limitation that may have prevented the observation of elevated IL-6 levels associated with RSHIs. There were no changes observed in TNF-a concentrations over time between groups (Figure 5D–F). Future work should examine the relationship between RSHIs and markers of acute inflammation.

Although statistically significant results correlating DHA+EPA plasma concentrations and NfL serum concentrations were not observed in the current study, there was a non-significant trend in participants from the treatment group with higher circulation DHA+EPA levels correlated with lower NfL serum concentrations (Figure 6). Even with DHA+EPA supplementation at 3.4 g/d 5 days a week, plasma concentrations and levels peaked at 21 weeks and slowly declined back to baseline by week 33, possibly due to non-compliance toward the end of the treatment protocol and the football season. The lack of statistical significance in these findings may have been impacted by insufficient plasma DHA+EPA levels and/or our small sample size.

The form of DHA+EPA provided may be another consideration. It is unclear in the current study whether DHA+EPA, provided in the form of ethyl esters, impacted circulating concentrations of DHA known to be capable of crossing the blood–brain barrier (BBB) and enriching brain parenchyma. DHA is the most abundant HUFA in the brain; however, DHA biosynthesis of the 18-carbon precursor alpha-linolenic acid (ALA) by brain endothelium is thought to be minimal. Consequently, DHA within circulating pools is thought to be vital in providing the brain with DHA. To enrich the brain, DHA must cross the BBB. Mechanisms of transport include passive diffusion of unesterified DHA [46] and active transport of lysophosphatidylcholine-DHA [46,47]. Future research utilizing sophisticated technologies such as lipidomic analysis may offer insight as to whether supplementation with ethyl esters or other n−3 HUFA forms (triglycerides, phospholipids, etc.) provides DHA+EPA in forms that more directly impact RSHIs.

## 5. Conclusions

Football is a sport that requires rapid acceleration, deceleration, and directional changes and includes head and body impacts in every game for some positions. This study highlights the impact of these practices on NfL, a biomarker of axonal integrity, and suggests disproportionate risk by start status and position. Although DHA+EPA supplementation did not decrease NfL, statistically significant neuroprotection from DHA+EPA could have been seen with a larger sample size or if NfL levels had been higher during the season. Alternatively, it is not clear that DHA+EPA was provided in a form that impacts brain tissue (e.g., phospholipids) or inflammation. Larger supplementation studies that investigate a variety of n-3 HUFA-containing lipid molecular species that may efficiently cross the blood–brain barrier may help identify more efficacious neuroprotective strategies.

## Figures and Tables

**Table 1:** Frequently reported adverse events by treatment group.

Adverse Event	Placebo (n = 19)	Treatment (n = 19)
Any, N (%)	10 (52)	12 (63)
>2 participants, N, (%)		
Respiratory infection	9 (47)	3 (15)
Acne	1 (5)	2 (10)
GI distress (stomach upset)	0 (0)	3 (15)
Halitosis and eructation	0 (0)	4 (21)
Concussion *	1 (5)	2 (10)
Serious AEs, N = 0 (0%)		

* Participants were removed from the study, but all returned to play within 3 weeks.

**Table 2:** ARA:DHA and ARA:EPA ratios by group and time.

	Baseline	Week 8	Week 12	Week 17	Week 21	Week 26	Week 33
ARA:DHA ratio treatment	7:1	3:1	3:1	3:1	3:1	4:1	5:1
ARA:DHA ratio placebo	5:1	5:1	5:1	5:1	5:1	6:1	5:1
ARA:EPA ratio treatment	23:1	6:1	8:1	6:1	8:1	11:1	21:1
ARA:EPA ratio placebo	19:1	20:1	21:1	16:1	18:1	17:1	16:1

Ratios in the treatment group were reduced from baseline values. DHA, docosahexaenoic acid; ARA, arachidonic acid; EPA, eicosapentaenoic acid.

## Data Availability

All deidentified data are available at ClinicalTrials.gov #NCT0479207.

## Supplementary Materials

The following supporting information can be downloaded at: https://www.mdpi.com/article/10.3390/nu14102139/s1, Table S1: Fatty acid profile of fish oil supplement and placebo; Table S2 Mean plasma fatty acid concentrations as a percent of the total by treatment and time.
