# Supplementary material for: Effects of Fish Oil on Biomarkers of Axonal Injury and Inflammation in American Football Players: A Placebo-Controlled Randomized Controlled Trial
Source: Nutrients. 2022 May 20;14(10):2139. doi: 10.3390/nu14102139 (PMC9146417; doi:10.3390/nu14102139)
Supplement: Supplementary file 1 [file nutrients-14-02139-s001.zip › Nutrients_supplemental_table S1.pdf]

Table S1. Fatty acid profile of fish oil supplement and placebo.

Figure .	DHA+EPA (% total)	DHA+EPA (mg FA/g oil)*	Placebo (% total)	Placebo (mg FA/g oil)*
Myristic (C14:0)	0.610		0.000	
Palmitic (C16:0)	1.710		5.647	
Palmitoleic (C16:1)	0.901		0.000	
Stearic (C18:0)	1.138		3.075	
Oleic (C18:1n9cis)	1.090	8.3	78.170	713
C18:1n11cis	0.298		0.858	
LA (C18:2n6cis)	0.000	0	12.976	14.2
GLA (C18:3n6)	0.381		0.000	
ALA (C18:3n3)	0.000		0.000	
Stearidonic (C18:4n3)	0.212		0.000	
Arachidic (C20:0)	0.734		0.000	
C20:1n9	1.483		0.000	
C20:2n6	0.320		0.000	
DGLA C20:3n6	0.365		0.000	
ARA (C20:4n6)	1.500		0.000	
(C20:4n3)	1.080		0.000	
EPA (C20:5n3)	22.000	170.5	0.000	0
Behenic (C22:0)	0.630		0.000	
Euricic (C22:1n9)	0.781		0.000	
Arsenic (C22:4n6)	0.561		0.000	
C22:5n6	2.010		0.000	
DPA (C22:5n3)	6.790		0.000	
DHA (C22:6n3)	53.440	407	0.000	0
C24:1n9	1.5830		0.223	
Total	100		100	

*Fatty acid concentrations (mg FA/g oil) were used to calculate daily supplement dose. FA fatty acid; LA linoleic acid; GLA, γ-Linolenic acid; ALA, $\boldsymbol{\alpha}$-linolenic acid; DGLA, dihomo-γ-linolenic acid; ARA, arachidonic acid; EPA, eicosapentaenoic acid, DPA, docosapentaenoic; DHA, docosahexaenoic acid. Fatty acid composition was determined as described in section "*Plasma fatty acid characterization*".
