# Supplementary material for: Effects of Fish Oil on Biomarkers of Axonal Injury and Inflammation in American Football Players: A Placebo-Controlled Randomized Controlled Trial
Source: Nutrients. 2022 May 20;14(10):2139. doi: 10.3390/nu14102139 (PMC9146417; doi:10.3390/nu14102139)
Supplement: Supplementary file 1 [file nutrients-14-02139-s001.zip › Nutrients_supplemental_table S2.pdf]

Table S2. Mean plasma fatty acid concentrations as a percent of total by treatment and time.

		Timepoint 1		Timepoint 2		Timepoint 3		Timepoint 4		Timepoint 5		Timepoint 6		Timepoint 7	
**FA**	**Common Name**	**Treatment**	**Placebo**	**Treatment**	**Placebo**	**Treatment**	**Placebo**	**Treatment**	**Placebo**	**Treatment**	**Placebo**	**Treatment**	**Placebo**	**Treatment**	**Placebo**
C14:0	Myristic	0.75	0.66	0.67	0.82	0.80	0.84	0.66	0.81	0.69	0.72	0.72	0.88	0.73	0.90
C15:0	Pentade-canoic	0.21	0.20	0.23	0.24	0.21	0.21	0.21	0.21	0.21	0.20	0.20	0.21	0.20	0.23
C15:1	n-Penta-decanoic	0.10	0.11	0.11	0.12	0.11	0.12	0.11	0.12	0.09	0.12	0.10	0.11	0.08	0.11
C16:0	Palmitic	20.38	20.62	20.66	21.35	20.57	20.58	20.26	20.38	19.95	20.35	20.23	21.08	20.24	21.66
C16:1	Pal-mitoleic	0.96	0.83	0.97	0.88	0.88	0.92	0.76	0.82	0.90	0.75	0.90	0.96	0.94	0.86
C17:1	Heptade-canoic	0.12	0.14	0.18	0.19	0.15	0.18	0.13	0.16	0.14	0.14	0.11	0.16	0.13	0.15
C18:0	Stearic	7.61	7.32	7.42	7.48	7.24	7.70	7.43	7.48	7.59	7.51	7.91	7.55	7.37	7.12
C18:1cis	Oleic	19.01	18.08	18.35	19.56	17.95	20.13	17.39	19.50	19.09	18.53	18.28	19.10	18.99	17.58
C18:1n7	Vaccenic	1.11	1.14	1.02	1.15	0.90	0.94	1.13	0.96	0.97	1.16	1.09	1.04	1.01	1.05
C18:2n6	LA	34.50	35.22	32.64	32.41	34.96	32.84	35.46	34.34	34.52	34.96	35.36	33.46	35.56	34.55
C18:3n3	ALA	0.74	0.80	0.59	0.61	0.78	0.66	0.67	0.76	0.63	0.72	0.69	0.84	0.89	0.83
C18:3n6	GLA	0.37	0.35	0.28	0.35	0.30	0.40	0.31	0.45	0.37	0.41	0.42	0.57	0.41	0.45
C18:4n3	Stea-ridonic	0.07	0.06	0.06	0.08	0.08	0.07	0.04	0.08	0.07	0.04	0.08	0.06	0.07	0.06

C20:1	Ei-cosenoic	0.16	0.13	0.15	0.14	0.17	0.16	0.15	0.15	0.16	0.16	0.15	0.14	0.17	0.14
C20:2	Eicosadi-enoic	0.30	0.29	0.27	0.29	0.27	0.31	0.27	0.31	0.28	0.30	0.29	0.31	0.30	0.30
C20:3n3	Eico-satetra-noic	0.06	0.12	0.08	0.12	0.11	0.11	0.08	0.10	0.07	0.11	0.06	0.12	0.05	0.13
C20:3n6	DGLA	1.26	1.23	1.28	1.14	1.16	1.28	1.23	1.40	1.20	1.32	1.22	1.34	1.22	1.36
C20:4n6	ARA	8.83	8.85	8.76	9.23	7.96	8.66	7.82	8.20	7.91	8.75	7.81	8.41	8.08	8.55
C20:5n3	EPA	0.38	0.47	1.53	0.46	1.02	0.42	1.30	0.50	0.97	0.49	0.72	0.49	0.39	0.53
C22:5n3	DPA-n3	0.46	0.48	0.52	0.47	0.45	0.47	0.45	0.47	0.46	0.47	0.41	0.46	0.44	0.50
C22:5n6	DPA-n6	0.19	0.19	0.12	0.23	0.09	0.22	0.09	0.20	0.12	0.20	0.07	0.21	0.13	0.21
C22:6n3	DHA	1.29	1.66	3.08	1.68	2.75	1.67	2.88	1.57	2.55	1.60	2.14	1.44	1.59	1.70

Significant differences in plasma concentrations across time and group were seen. FA, fatty acid; LA linoleic acid; ALA, **α**-linolenic acid; GLA, γ-Linolenic acid; DGLA, dihomo-γ-linolenic acid; ARA, arachidonic acid; EPA, eicosapentaenoic acid, DPA-n3, docosapentaenoic-n3; DPA-n6, docosapentaenoic-n6; DHA, docosahexaenoic acid
